# ChIP‐MS in Plant Systems: Mapping the H3K27ac Proteome During the Greening Process

**DOI:** 10.1111/ppl.70797

**Published:** 2026-02-18

**Authors:** Alexis Brun, Marti Quevedo, Luis A. Sterling, Dick H. W. Dekkers, Jeroen Demmers, Elton Paul Hudson, Åsa Strand

**Affiliations:** ^1^ Umeå Plant Science Centre, Department of Plant Physiology Umeå University Umeå Sweden; ^2^ Proteomics Center, Erasmus University Medical Center Rotterdam the Netherlands; ^3^ School of Engineering Sciences in Chemistry, Biotechnology and Health, Science for Life Laboratory KTH – Royal Institute of Technology Stockholm Sweden

**Keywords:** chromatin, histone modifications, MS, photosynthesis

## Abstract

We have established a method for chromatin immunoprecipitation coupled to mass spectrometry (ChIP‐MS) in *Arabidopsis thaliana* . We demonstrate its utility by investigating proteins associated with histone H3 lysine 27 acetylation (H3K27ac), a key epigenetic mark regulating photosynthesis‐associated nuclear genes (*PhANGs*) during chloroplast development and establishment of photosynthesis. Purification of chromatin‐associated proteins from light‐grown Arabidopsis cell cultures identified 66 proteins associated with H3K27ac that met the selection criteria in the two replicate experiments: (i) 2‐fold change in relation to IgG, (ii) at least two unique peptides, and (iii) relevant biological annotations. The identified proteins included chromatin remodelers, chromatin regulators and transcription factors with potential roles in H3K27ac deposition. To evaluate the physiological role of the candidates associated with the H3K27ac mark, we developed a rapid and reproducible phenotyping method based on controlled light scanning to determine chlorophyll accumulation in mutant seedlings. We complemented with pigment quantification and analysis of photosynthesis‐associated nuclear genes (*PhANGs)* expression. Several mutants displayed altered greening, pigment accumulation, or affected photosynthetic gene expression consistent with a role during chloroplast development. Notably, *chr11, chr17*, and *atpds5a* mutants showed impaired pigment accumulation and reduced expression of *PhANGs*, whereas *hmgb4* and *mbd10* mutants exhibited increased greening and induction of *PhANGs*. Together, these findings establish ChIP‐MS as a robust approach to identify histone mark‐associated proteins in plants and provide a first set of candidate regulators of H3K27ac during chloroplast biogenesis. This technical advance opens new possibilities to discover chromatin‐based regulation of plant development and environmental responses.

## 1 Introduction

Our understanding of transcriptional regulation in plants has increased considerably over the past decade, largely due to advances in whole‐genome sequencing. Techniques such as Chromatin Immunoprecipitation followed by sequencing (ChIP‐seq) have enabled the mapping of transcription factor binding sites and revealed the genome‐wide distribution of regulatory features, including histone modifications—commonly referred to as histone marks—that serve as readouts of the chromatin state (Mikkelsen et al. 2007). For instance, acetylation of histone H3 is a hallmark of open, accessible chromatin and is typically associated with active genes and enhancers. In contrast, specific methylation marks—such as trimethylation of lysine 27 on histone H3 (H3K27me3)—are characteristic of repressed regions and silencers. Although numerous factors have been linked to specific plant histone marks (Le et al. 2025), there remains significant potential to uncover novel trans‐regulatory proteins associated with these modifications—and, more importantly, to elucidate their biological roles in plant physiology.

The common approach investigating chromatin factors typically begins with characterizing their genomic occupancy and then linking this information to known chromatin features, such as specific histone marks. While this strategy provides broad insights into factor localization, it offers limited information about the molecular context in which these factors operate. An alternative, less common strategy reverses this perspective: starting from defined genomic regions or chromatin features and tracing back to the associated proteins. For instance, focusing on histone marks such as H3K27ac and identifying their protein–protein interactors. In mammalian systems, this has been achieved through Chromatin Immunoprecipitation coupled with mass spectrometry (ChIP‐MS), which enabled the purification and identification of both known and novel proteins associated with specific chromatin marks (Engelen et al. 2015; Ji et al. 2015). Proteomic studies in Arabidopsis have thus, so far, concentrated mainly on global profiling of histone modifications by mass spectrometry (Scheid et al. 2022) or on locus‐specific purification (Lynn et al. 2025). However, the integration of ChIP with MS to investigate the protein environment of individual histone marks remains largely unexplored in plants.

As in animals, the dynamics of histone marks in plants are key indicators of transcriptional regulatory mechanisms that shape both development and stress responses. The balance between histone acetylation and methylation at specific loci regulates diverse processes, including dormancy and germination, light and shade perception, photosynthetic establishment, the vegetative‐to‐floral transition, and responses to environmental stress (Charron et al. 2009; Bouyer et al. 2011; He 2012; Liu et al. 2013; Nonogaki 2014; Lämke and Bäurle 2017). We recently characterized chromatin dynamics during the establishment of photosynthesis in Arabidopsis and identified H3K27ac as a key mark that integrates retrograde plastid signals into the nuclear epigenome (Quevedo et al. 2025). Forward genetic approaches further revealed transcription factors, such as members of the Golden2‐Like (GLK) family, associated with H3K27ac deposition at photosynthesis‐associated nuclear genes (*PhANGs*). However, the proteome linked to acetylated nucleosomes remains largely unexplored.

Here, we successfully adapted ChIP‐MS to plant systems, uncovering novel chromatin interactors of acetylated H3, including chromatin remodelers, core components of the transcriptional machinery, transcription factors, and additional chromatin regulators. To efficiently prioritize candidates from the high‐throughput datasets typical of MS analyses, we also developed a rapid phenotype screening strategy focused on accumulation of photosynthetic pigments. By combining greening image scans with quantification of *PhANG* transcript levels in mutant lines, we validated the involvement of several newly identified chromatin‐associated proteins in the regulation behind the establishment of photosynthesis. Thus, we present a protocol for ChIP‐MS for identifying novel regulators associated with histone modifications. This technical advance provides a tool to discover the details of chromatin‐based regulation in plant biology.

## 2 Material and Methods

### 2.1 ChIP‐MS Procedure

#### 2.1.1 Chromatin Preparation

Chromatin preparation was achieved by combining protocols from ChIP‐MS in mammals (Engelen et al. 2015) and ChIP in Arabidopsis (Quevedo et al. 2025), with the following modifications. An Arabidopsis single cell suspension culture was grown in the light for 7 days until full photosynthetic establishment (Dubreuil et al. 2018; Quevedo et al. 2025). At this point, at least 50 g of cells per replicate were recovered by filtration and dry snap‐frozen in liquid nitrogen. To ensure homogenic tissue disruption, frozen cells were processed with a cryogenic grinder always in liquid nitrogen. Next, small batches of 5 g were processed individually in parallel. Each aliquot of 5 g of ground powder was resuspended in 50 mL of MC buffer (10 mM sodium phosphate, pH 7, 50 mM NaCl and 1% sucrose, 2 mM PMSF) and double crosslinked. In detail, ground cells resuspended in MC buffer were incubated with 2 mM of ethyleneglycol‐bis‐succinimidyl‐succinate (EGS, Thermo‐scientific) in constant agitation at room temperature for 30 min, and then supplemented with 0.75% formaldehyde for an additional 10 min (total EGS incubation 40 min). Crosslinking was stopped with a final concentration of 125 mM Glycine for 10 min. Crosslinked powder was washed twice with chilled MC buffer and immediately processed for chromatin isolation using our adapted Arabidopsis ChIP protocol (Lichtenthaler and Buschmann 2001; Warren 2008). Briefly, after MC washes, the pellet was resuspended in M1 buffer (10 mM sodium phosphate, pH 7, 0.1 M NaCl, 1 M 2‐methyl 2,4‐pentanediol, 1% sucrose, 10 mM β‐mercaptoethanol and 2 mM PMSF) supplemented with 5 mM DTT and passed through a Miracloth layer. After centrifugation, pellet was washed at least four times with M2 buffer (10 mM sodium phosphate, pH 7, 0.1 M NaCl, 1 M 2‐methyl 2,4‐pentanediol, 1% sucrose, 10 mM β‐mercaptoethanol, 10 mM MgCl_2_, 0.5% Triton X‐100, and 2 mM PMSF) and 1 time with M3 buffer (10 mM sodium phosphate, pH 7, 0.1 M NaCl, 1% sucrose, 10 mM β‐mercaptoethanol, and 2 mM PMSF). Clean nuclei were resuspended in sonication buffer (10 mM sodium phosphate, pH 7, 0.1 M NaCl, 0.5% Sarkosyl, 10 mM EDTA and 2% SDS). Chromatin shearing was achieved with a focused‐ultrasonicator (Covaris S2 system). DNA from a small aliquot of each chromatin extract was isolated, quantified and tested to contain 100–500‐bp fragments.

#### 2.1.2 Chromatin Immunoprecipitation

From the starting 50 g of cells for one experiment, chromatin was split for two pull‐downs, one with anti‐H3K27ac and one with IgG. To prevent immunoglobulin elution and subsequent interference with the MS analysis, 50 μg of antibody (H3K27ac from Abcam ab4729 and IgG from Agrisera AS10916) were crosslinked to 500 μL Protein A magnetic bead solution (15 mg beads, Life Technologies) with Dimethyl Pimelimidate (Sigma). Crosslinked antibody–bead complexes were washed in IP buffer (50 mM HEPES, pH 7.5, 150 mM NaCl, 5 mM MgCl2, 1% Triton X‐100, and 0.05% SDS) and subsequently blocked with 0.5 mg mL^−1^ BSA (New England Biolabs) for 1 h. For each immunoprecipitation, chromatin was split into 5 aliquots in Eppendorf tubes and sonication buffer was diluted with IP buffer to reach a final concentration of 0.1% SDS and supplemented with Complete protease inhibitor cocktail (Roche). Chromatin was incubated with antibody–bead mixture while rotating overnight at 4°C, washed five times for 5 min in RIPA buffer (50 mM HEPES‐KOH (pH 7.6), 500 mM LiCl, 1 mM EDTA, 1% NP‐40, 0.7% Na‐deoxycholate). In the last wash, bead aliquots from the same *Immunoprecipitation* were pooled in one eppendorf and boiled for 35 min at 95°C in 2 × SDS (Laemmli) sample buffer (100 mM Tris–HCl (pH 6.8), 200 mM DTT, 4% SDS, 20% Glycerol, 0.2% Bromophenol blue), centrifuged at max speed and supernatant was transferred to a fresh tube. ChIP‐MS samples were run on 10% precast SDS–PAGE gels (NuPage Invitrogen) and stained with colloidal Coomassie stain (Invitrogen). Gel lanes were sliced, in‐gel digested with trypsin to yield peptides and proteins identified by analyses on an LQT‐Orbitrap mass spectrometer (Thermofisher). ChIP‐MS inclusion criteria: Two independent ChIPs were performed for H3K27ac and for IgG as control and analyzed by MS (Table S1). Candidates that were only present in both replicates and with a 2 LFQ intensity fold enrichment over IgG were selected.

### 2.2 ChIP‐Seq Analysis

Bigwigs (Kent et al. 2010) were downloaded from publicly available datasets as follows. Histone PTMs (Quevedo et al. 2025); DEK3, GSM3137195 (Brestovitsky et al. 2019); CHR11, GSM4141907 (Luo et al. 2020); HD2C, GSM2882805 (Chen et al. 2018); TPL, GSM6552664 (Zhong et al. 2023); and TPR1, GSE149316 (Griebel et al. 2023). Tracks were visualized in IGV (Robinson et al. 2011) exported as pdf and formatted in Illustrator. Heatmaps were constructed using bigwigs with deepTools (Ramírez et al. 2016) and bed peak files from our previous study (Quevedo et al. 2025), keeping the same scale for each factor to compare between H3K27ac and H3K27me3 regions.

### 2.3 Growth Conditions

Arabidopsis cell culture was grown as in Dubreuil et al. (2018). Samples were collected by filtration. Sample material from multiple flasks was pooled together before grinding. Mutant T‐DNA insertion lines were obtained from the Arabidopsis Stock Centre. The lines were genotyped and confirmed homozygous for the insertion (Figure S2). Primers used for genotyping have been designed using the T‐DNA Salk tool (http :// signal.salk.edu/tdnaprimers.2.html) and are listed in Table S2. Arabidopsis seeds were surface‐sterilized with ethanol and sodium hypochlorite and stratified at 4°C for 3–5 days. Seedlings were grown on MS medium (0.5× Murashige and Skoog (MS) basal salt; 0.05% MES; pH 5.7) containing 1% plant agar. Seedling (de)etiolation consisted in a first exposure of 3 h of light after seed stratification for 2 days at 4°C, followed by 3 days of darkness growth at 21°C. Dark‐grown seedlings were collected in the dark under a green light or moved to a LED continuous light cabinet (100 μmol photons m^−2^ s^−1^), harvested at specific time‐points and snap frozen in liquid nitrogen.

### 2.4 Fast Screening of Greening Phenotype

#### 2.4.1 Image Acquisition

At the indicated time points (0, 6, 12, and 24 h after transfer to light), entire agar plates containing seedlings were scanned using an EPSON Expression 12000XL flatbed scanner equipped with a built‐in transparency unit to ensure uniform illumination. Scans were acquired in 24‐bit RGB color, saved as uncompressed TIFF format images at a resolution of 2400 ppi, with output scaling fixed to 120 × 120 mm to ensure reproducibility across time points and plates. Each plate contained seedlings of the same genotype. The scanner light source provided consistent illumination, avoiding the need for DSLR cameras or external lighting calibration.

#### 2.4.2 Image Pre‐Processing

Image processing was performed in ImageJ (v.1.54p, Java 1.8.0_322, 64‐bit). The workflow consisted of two automated steps with an in‐house ImageJ macro; Figure S4:

1. B&C adjustment: prior to measurement, brightness/contrast levels were adjusted within the range 50–150 for each RGB channel.
2. Channel separation: RGB images were split into individual channels, and only the 8‐bit green channel was retained for further analysis as a proxy for chlorophyll accumulation.

#### 2.4.3 Quantification of Cotyledon Greening

For each image, Regions of Interest (ROIs) were manually drawn around cotyledons. Only seedlings growing in the same focal plane and with cotyledons fully visible were selected to ensure consistency. A minimum of *n* ≥ 25 seedlings per condition was analyzed. For each ROI, the mean grey value was extracted and converted to a relative greening index by subtracting the measured mean grey value from the maximum grey value (255). This provided a normalized value where higher scores correspond to increased greening.

#### 2.4.4 Statistical Analysis

Data were compiled in GraphPad Prism 10. For each genotype, the distributions of greening values across all time points (0, 6, 12, 24 h) were compared with those of the wild type (Col‐0). Statistical significance was assessed using one‐way ANOVA, followed by Dunnet's post hoc test for multiple comparisons. At least three independent biological replicates were included. Results are reported as mean ± SEM. Representative examples of greening kinetics are shown in Figure S5.

### 2.5 Apical Hook Determination and Quantification of Photosynthetic Pigments

Apical hook angle was measured using ImageJ (Fiji) by determining the angle between two lines: one line is drawn along the hypocotyl; another is drawn perpendicularly between the two cotyledons. A complete ideal opening corresponds to 180°. A fully closed apical hook corresponds to 0°–20°. Extraction of pigments was performed in two rounds to ensure complete extraction by adding 250 μL of cold methanol to 25 mg of ground frozen sample, mixed, and left on ice in darkness for 5 min. Next, samples were centrifuged (5 min, 12,000 × *g* 14,000 rpm, 4°C). From the pooled 500, 400 μL was used to fill two wells (as technical replicates) in a 96‐well microplate (83.3924, SARSTEDT). A470, A652, A665, and A750 absorbances were read with a SpectraMAX 190 microplate reader. Quantification of pigment content was achieved following the protocol adapted from (Dubreuil et al. 2018). First, the absorbance was corrected for a volume of 200 μL MeOH in microplates. Chlorophyll A: Corrected A652c = (A652 − A750)/0.58; Chlorophyll B: Corrected A665c = (A665 − A750)/0.58; Carotenoids: Corrected A470c = (A470 − A750)/0.58. Afterward, pigment concentration was calculated: ChlA (μg mL^−1^) = [−6.5079 × A652c] + [16.2127 × A665c]; ChlB (μg mL^−1^) = [32.1228 × A652c] − [13.8255 × A665c]; Carotenoids (μg mL^−1^) = [1000 × A470c] − [1.63 × ChlA]  − [104.96 × ChlB]/221. Finally, the total amount of pigments in μg mg^−1^ of fresh weight was recalculated. A750 was used as a blank.

### 2.6 RT‐qPCR

Total RNA was extracted from frozen ground powder from Arabidopsis seedlings with the RNeasy Kit following the manufacturer's instructions (QIAGEN). Extracted RNA was treated with DNase I (Thermo Scientific). Complementary DNA (cDNA) was synthesized using iScript cDNA Synthesis Kit (Bio‐Rad) following the manufacturer's instructions. qPCR reactions were performed using iQ SYBR Green Supermix (Bio‐Rad) on CFX96 or 384 Real‐Time System (Bio‐Rad). Relative gene expression was normalized to the mean of two control targets, *UBC* and *EF1‐α*, as they do not significantly change in expression in response to dark–light shifts, and relative values were calculated to each control condition. Data analysis was done using CFX manager (Bio‐Rad) and LinRegPCR software (Ramakers et al. 2003). Primers used for qPCR are listed in Table S3.

## 3 Results

### 3.1 ChIP‐MS Identifies Novel Components Associated With Histone Acetylation

**FIGURE 1:**
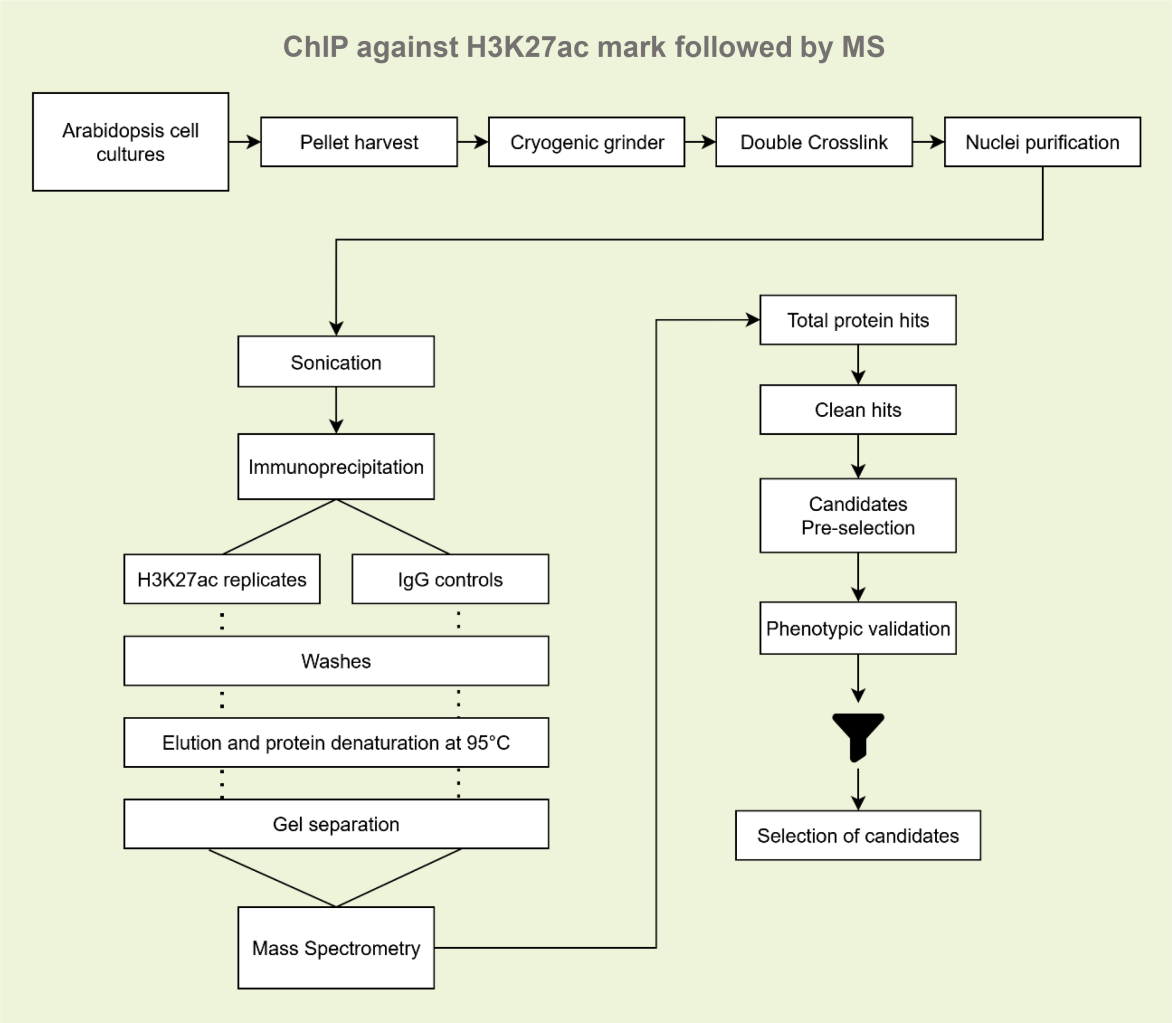
ChIP‐MS procedure. Schematic representation of the established ChIP‐MS method step by step. After harvesting the cell culture, the cell pellet was flash frozen and ground in liquid nitrogen. Double crosslink: Cells were resuspended and crosslinked with ESG and formaldehyde. After stopping the reaction with glycine, clean nuclei were isolated and sonicated to obtain chromatin. Chromatin shearing was checked by electrophoresis before the pull‐downs (immunoprecipitation [IP]). Equal amounts of chromatin were used to immunoprecipitate, using either the specific antibody for the H3K27acetylation mark or IgG antibody with covalently coupled magnetic beads. Following washing steps, samples from the same replicate IP were pooled and boiled. Clean samples were run on 10% acrylamide gels, bands cut, digested with trypsin and proteins identified by Mass Spectrometry. Two experimental replicates were run in this study.

We performed two independent ChIP experiments using an anti‐H3K27ac antibody and processed the immunoprecipitates for mass spectrometry‐based proteomic analysis (Figure 1). Adaptation of the ChIP‐MS protocol from animal embryonic stem cells (ESCs) to plant cells demanded a number of fundamental modifications in view of unavoidable structural and biochemical differences between the two model cells. We chose a plant suspension cell culture as a homogenous, highly scalable, easy‐to‐collect and reproducible sample. To reach the chromatin amounts of ~300 × 10^6^ attached ESCs, we aimed for ~50 g of Arabidopsis cell culture, to overcome limitations such as differences in size between animal and plant cells, the large vacuolar compartment in plant cells, and less accessible chromatin per gram of tissue in plants compared to animal cells (Deal and Henikoff 2011). To enable efficient disruption of cells, instead of lysing entire cells in plates as in animals, dry plant cells were frozen in liquid nitrogen, removing excessive water, and cryogenically ground to powder prior to crosslinking; the mechanical disruption was needed to rupture the hard cellulose cell wall and to allow uniform access of reagents to nuclei (Canton et al. 2022). We noticed that grinding of already crosslinked material or with excessive water content severely impaired complete disruption and yielded less nuclei. Thus, the crosslinking procedure was also changed: already ground plant tissue was thawed at room temperature in crosslinking solution containing 2 mM EGS for 30 min with the addition of 0.75% formaldehyde for 10 min, after which it was quenched using 125 mM glycine for 10 min, always in the presence of protease inhibitors. The use of EGS, which has a longer spacer arm than that of DSG, may improve the crosslinking between proteins, while the lower concentration and shorter treatment with formaldehyde avoided excessive crosslinking in ground nuclei suspensions, known to reduce recovery of plant nuclei. Nuclei isolation also required significant adaptations: the HEPES‐buffered LB buffers (LB1–LB3) used with animal cells were replaced by a set of plant‐specific MC/M1/M2/M3 buffers with added osmotic stabilizers (sucrose, 2‐methyl‐2,4‐pentanediol), reducing agents (β‐mercaptoethanol, DTT), and detergents, followed by low‐speed repeated washing and Miracloth filtration to remove cell wall debris, plastids, and phenolic pollutants—components absent in animal cells that otherwise reduce yield and compromise chromatin integrity (Deal and Henikoff 2011). Chromatin shearing was also changed from probe sonication (Soniprep) in animal extracts to concentrated ultrasonication (Covaris) in plant extracts, which highly increased shearing efficiency and consistency among different batches. For immunoprecipitation, plant chromatin was diluted to 0.1% SDS and also supplemented with protease inhibitors, unlike direct use of LB3‐solubilized chromatin in animals, to counteract the excessive endogenous protease activity inherent in plant extracts (Ramirez‐Prado et al. 2021). Finally, while downstream washes, SDS–PAGE (Figure S1), and Orbitrap MS analysis were conceptually identical, we used inclusion criteria based on detection in both replicates and greater than or equal to twofold LFQ intensity versus IgG. Collectively, these alterations bypassed plant‐specific challenges such as cell walls, secondary metabolites, and enhanced proteolytic activity. It enabled efficient recovery of chromatin protein complexes from Arabidopsis cells for further Mass Spectrometric analysis.

**FIGURE 2:**
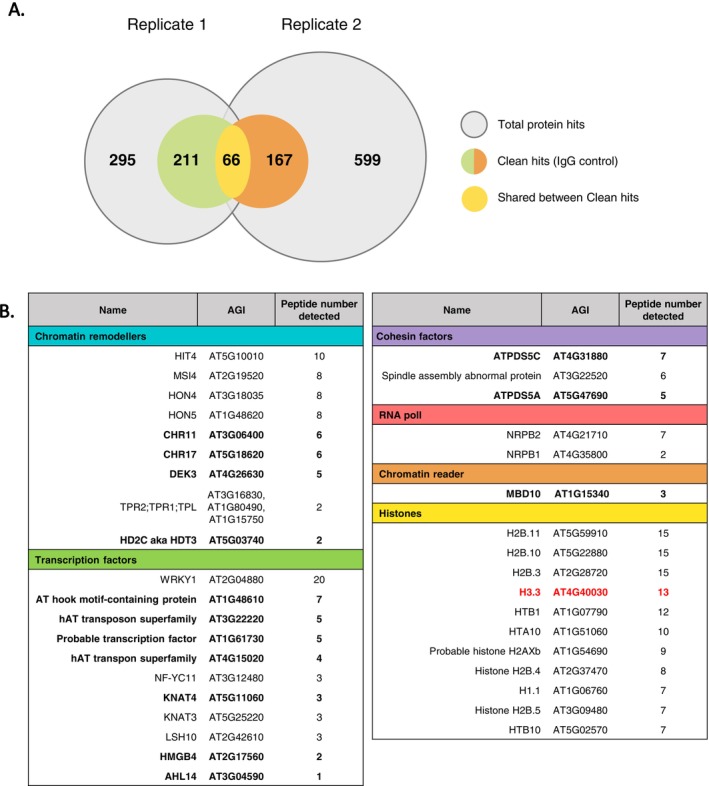
Proteins identified from ChIP‐MS. (A) The Venn diagram displays clean hits filtered with results from the IgG control for replicates 1 and 2. The overlap between the two replicate experiments is displayed. (B) Diagram of selected candidates detected by Mass Spectrometry grouped into functional categories. Histone H3.3 is the bait (H3.3K27ac marked in red). The candidates from which we acquired T‐DNA lines to perform the phenotypic validation are indicated in bold.

Fold‐enrichment values were calculated relative to a control (IgG) for each replicate. We selected proteins that met three criteria: (i) a fold change above twofold in both replicates, (ii) consistent detection (e.g., at least two unique peptides across both experiments), and (iii) relevant biological interactions (e.g., GO terms related to chromatin regulation or transcription), and we discarded common proteomic contaminants as in Mellacheruvu et al. (2013). Proteins meeting these quantitative, annotation‐based, and literature‐supported criteria were designated as candidate H3K27ac‐associated proteins. A final list of 66 clean hits was retrieved (Histone H3.3 included) (Figure 2A; Table S1) and candidate proteins were compiled once the criteria of biological relevance was met (Figure 2B). Our initial focus was on chromatin remodelers and transcription factors, and several candidates emerged with known or predicted roles in chromatin remodeling, 3D genome organization, or epigenetic regulation (Figure 2B). In the transcription factor category, some potentially interesting candidates were the following: AHL14 (AT3G04590) is a DNA‐binding helicase likely involved in chromatin remodeling through H3.3 deposition, influencing nucleosome positioning and transcription (Charron et al. 2009; Yun et al. 2012; Zhao et al. 2013). AT hook motif‐containing protein (AT1G48610) acts as an ATP‐dependent remodeler required for heterochromatin structure and DNA repair (Kotliński et al. 2017). NF‐YC11 (AT3G12480), also known as DDM1, promotes transposon silencing and heterochromatin formation via deposition of H2A.W (Siefers et al. 2009; Petroni and Tonelli 2011). LSH10 (AT2G42610) functions as a repressor by recruiting the histone deubiquitinase OTLD1 to target promoters (Vo Phan et al. 2023). KNAT4 (AT5G11060) is a homeobox transcription factor involved in ovule development and auxin responses, likely acting through the AS1/AS2/HIRA complex (Guo et al. 2008; Chen et al. 2023). Two further candidates correspond to hAT transposon‐like loci (AT3G22220, AT4G15020). Their roles remain unknown, but there are indications towards a role during responses to abiotic stress (Joly‐Lopez et al. 2017) and related hAT elements such as DAYSLEEPER have acquired essential functions in development (Bundock and Hooykaas 2005).

In the category of chromatin remodelers, we identified DEK3 (AT4G26630), a chromatin‐associated factor that binds histones and interacts with cohesin and topoisomerase, modulating accessibility and gene expression (Waidmann et al. 2014). CHR11 (AT3G06400) and CHR17 (AT5G18620) are ISWI‐type re‐modelers that organize nucleosome arrays to maintain chromatin structure (Li et al. 2014, 2017). HMGB4 (AT2G17560) belongs to the HMG family and bends DNA to facilitate access for regulators (Thomas and Travers 2001; Ueda and Yoshida 2010). HDT3/HD2C (AT5G03740) is a plant‐specific histone deacetylase that cooperates with HDA6 and SWI/SNF re‐modelers in ABA, salt, and heat stress regulation (Luo, Liu, et al. 2012; Luo, Wang, et al. 2012). ATPDS5A (AT5G47690) is a cohesin‐associated protein that shapes 3D genome architecture by limiting chromatin loop formation, a process linked to meiosis and DNA repair (Pradillo et al. 2015; Göbel et al. 2024). *MBD10* (AT1G15340) encodes a methyl‐CpG binding protein (methylated DNA reader) with roles in nucleolar dominance and ABA‐induced senescence, pointing to an epigenetic function in stress responses (Lang et al. 2015; Li et al. 2024).

**FIGURE 3:**
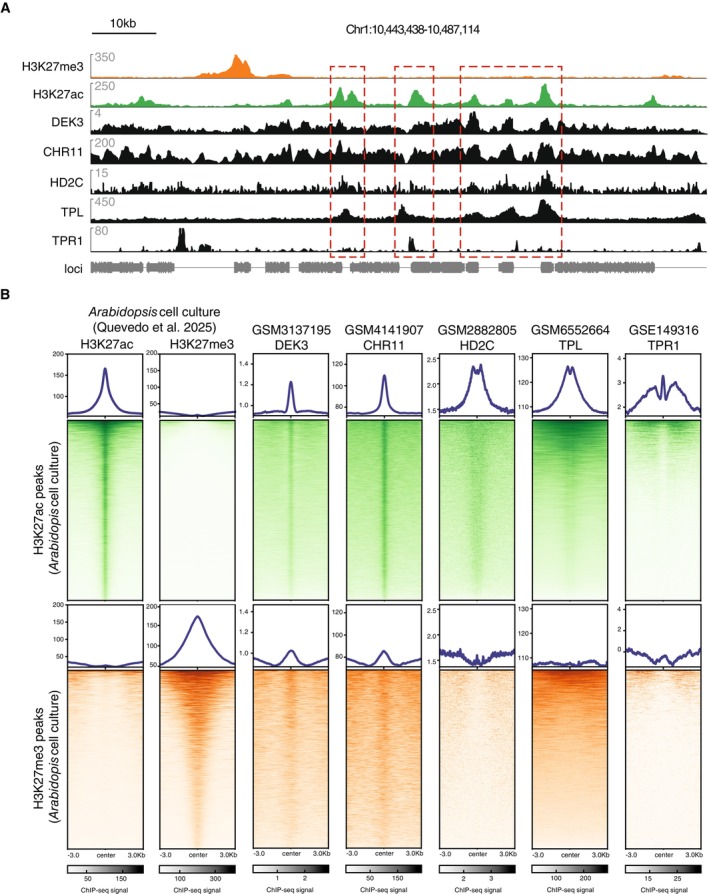
Genome‐wide occupancy of selected candidates. (A) Chromatin Immunoprecipitation (ChIP)‐sequencing(seq) visualization tracks of histone PTMs occupancy and H3K27ac ChIP‐MS interactors. A normalized ChIP‐seq signal is indicated on the *y*‐axis. A scale bar is indicated. (B) Heatmaps displaying the overlap between Day 7 Arabidopsis cell culture H3K27ac or H3K27me3 and published Arabidopsis seedling ChIP‐seq data of H3K27c interactors. The ChIP signal of each histone PTM is ranked according to H3K27ac peaks (greens) or H3K27me3 (oranges). At each heatmap, lines correspond to peaks which are sorted based on ChIP‐seq signal and displayed in a 6 kb window centered at the peak summit. The same scale is used to compare H3K72ac and H3K27me3 regions.

In order to validate the ChIP‐MS results, we evaluated the genome‐wide binding sites of 5 of our H3K27ac‐interactor candidates employing published ChIP‐seq datasets (Figure 3A). In detail, H3K27ac ChIP‐seq peaks from our cell culture were ranked by H3K27ac ChIP‐seq signal (Quevedo et al. 2025) and were represented in a genome‐wide heatmap. As expected, H3K27me3 signal was not present in these regions. Over these ranked regions, the ChIP‐seq signal for our candidates DEK3, CHR11, HD2C, TPL, and TPR1 displayed an enrichment for H3K27ac regions, either exactly at the center of H3K27ac (DEK3, CHR11) or surrounding the peak center (HD2C, TPL, TPR1) (Figure 3B upper green heatmaps). Our candidate proteins showed no binding or lower affinity occupancy for H3K27me3, proving specificity for H3K27ac (Figure 3B lower orange heatmaps). Taken together, these candidates from our ChIP‐MS dataset represent potential interactors with the acetylated chromatin context in Arabidopsis.

### 3.2 Fast Screening of Mutant Lines of Candidate Proteins for Greening Phenotype

**FIGURE 4:**
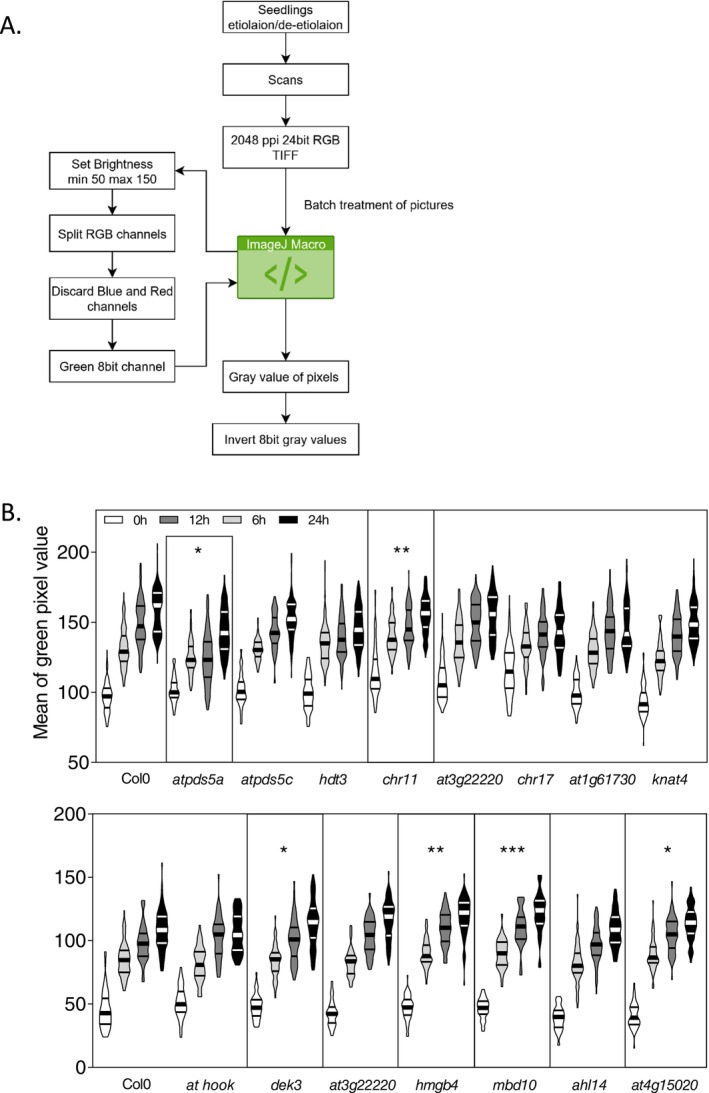
Fast screening method for mean green pixel value of Arabidopsis cotyledons. (A) A schematic overview of the screening method used to detect green accumulation in seedlings during de‐etiolation. (B) Violin plots of the mean green pixel values of cotyledons from mutants of the selected candidate proteins. Sample sizes range from 38 to 163 screened cotyledons for each mutant line and de‐etiolation time point. Three‐day‐old etiolated seedlings were exposed to constant white light for 6, 12, and 24 h. Significance was assessed using Kruskall–Wallis comparisons followed by Dunn post hoc test for each mutant line against Col‐0 [*p*‐value thresholds: *p* < 0.05 (*), < 0.01 (**), < 0.001 (***)]. Growth plates in the two presented plots were processed in separate batches, with their respective Col‐0 control. A shift in absolute mean green pixel values between plots is observed due to the use of a different black background during scanning for each batch.

In wildtype plants, H3K27ac is enriched at *PhANG* loci in a light and chloroplast‐signal dependent manner, leading to chlorophyll biosynthesis and accumulation in greening seedlings (Quevedo et al. 2025). Hence, to rapidly validate potential candidate proteins identified from the ChIP‐MS analysis (Figure 2B in bold), we obtained and confirmed homozygous T‐DNA mutant lines for the key candidates (Table S2; Figure S2). We obtained homozygous T‐DNA mutant lines for 14 candidate genes and screened mutant seedlings for a phenotype during the greening process. We started by assessing the accumulation of chlorophyll by a quantification of green levels in camera pictures. Traditional methods are based on the comparison of digital single‐lens reflex (DSLR) camera pictures. However, this approach has limitations: (1) colour comparison depends entirely on the light nature and intensity, a parameter difficult to consistently reproduce using an external illumination source and a camera; (2) the need to control white balance and to replicate its parameters over time and light sources; (3) the requirement of specialized training on camera operation; and (4) the significant time‐consumption needed to capture extensive amounts of pictures in large scale screens. We addressed these limitations by developing a novel method to scan seedlings with a controlled light source, to track the green colour of their cotyledons over time while increasing the number of individuals, allowing us to perform statistical tests on large populations of seedlings (Figures 4A, and S3–S5). This method is rapid, highly reproducible, easy to learn and does not necessitate any studio with controlled light sources or expensive DSLRs. The method is detailed in Figures S3 and S4 but can be briefly described as follows. Seedlings were grown in the dark (etiolated) for 3 days in vertical plates and placed under constant light. Seedlings were then scanned at 0 h (right after etiolation) and at 6, 12, and 24 h following constant light exposure. Scans were RGB TIFF files and the green color channel was used to measure cotyledons greening over time, with ImageJ open‐source software (Figures 4 and S5). For each genotype, the populations from four time points (0, 6, 12, 24 h) were compared to Col0 (wild type). Among the 14 candidate mutant lines tested, two genotypes showed significant decrease of green pixel value (*atpds5a and chr11*) and four presented a significant increase of green pixel value (*dek3*, *hmgb4*, *mbd10*, and *at4g15020*) (Figure 4B). After this initial screen we quantified the accumulation of photosynthetic pigments.

**FIGURE 5:**
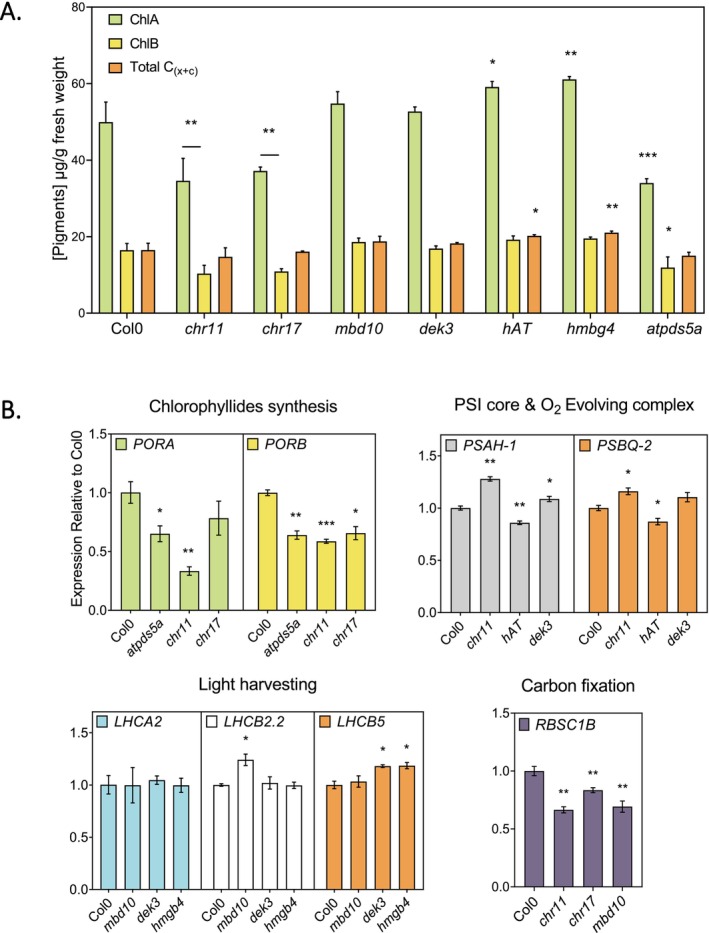
Quantification of photosynthetic pigment and *PhANGs* expression. (A) Bar plot representing quantification of the accumulation of photosynthetic pigments in the different mutant lines. Three‐day‐old seedlings grown in darkness followed by 12 h of white light (*n* = 3, independent biological replicates). Significance was assessed using ordinary one‐way ANOVA comparisons followed by Dunnet's post hoc test for each mutant line against Col‐0 [*p*‐value thresholds: *p* < 0.05 (*), < 0.01 (**), < 0.001 (***)]. (B) Expression of *PhANGs* was determined by RT‐qPCR using *UBC* and *EF1‐α* as housekeeping genes. Three‐day‐old seedlings grown in the dark were exposed to 12 h of white light (*n* = 3 independent biological replicates). Expression is presented relative to Col‐0. Statistical analysis has been performed by a two‐way ANOVA test followed by a Dunnet's post hoc test [*p*‐value thresholds: *p* < 0.05 (*), < 0.01 (**), < 0.001 (***)].

Three‐day‐old etiolated seedlings exposed to 12 h of constant light were analyzed and we selected *chr11*, *chr17*, *atpds5a, mbd10*, *hmbg4, dek3*, and *at3g22220* to assess their photosynthetic pigment concentration (Figure 5A). The *at3g22220* and *hmgb4* mutant lines showed a significant increase in chlorophyll *a* accumulation compared to Col0, whereas the *chr11*, *chr17*, and *atpds5a* mutant lines displayed a significant decrease in chlorophyll accumulation compared to Col0. Thus, the quantification of the chlorophyll content reproduces the results from the initial greening screen (Figures 4, 5, and S6). In addition to increased chlorophyll accumulation, *at3g22220* and *hmgb4* showed an increased accumulation of carotenoids. We also determined the opening of the cotyledons in response to light (Figure S7) and, in addition to a decrease in chlorophyll accumulation, *chr17* and *atpds5a* mutant lines displayed impaired cotyledon opening compared to Col0 in response to light (Figure S7).

### 3.3 Analysis of *PhANG* Expression in the Mutant Lines Indicates a Role for Some of the Identified Proteins in the Greening Process

H3K27ac mark deposition correlates with the induction of *PhANGs* expression (Quevedo et al. 2025). To refine the filtering of candidate proteins identified from the ChIP‐MS, we determined the expression of *PhANGs* in selected mutant lines. Three‐day‐old etiolated seedlings were exposed to 12 h of constant light and expression of *PhANGs* was determined by RT‐qPCR. The expression of 10 different genes involved in various aspects linked to photosynthesis and that are subject to H3K27 acetylation were measured: *PORA, PORB* (chlorophyllides synthesis), *LHCA2, LHCB2.2, LHCB5* (light harvesting), *PSAH1, PSBQ2* (PSI core and O_2_ evolving complex) and *RBCS1B* (carbon metabolism).

In the *mbd10*, *dek3*, and *hmgb4* mutant lines that presented an increase of green pixel value (Figure 4B), we found increased expression of *LHCB2.2* and *LHCB5* compared to Col0 (Figure 5B). The enhanced expression of *LHCB* suggests a potential role in the regulation of *PhANGs* for MBD10, DEK3, and HMGB4. DEK3 and HMGB4 have been linked to chromatin/histone regulation of transcription (Thomas and Travers 2001; Ueda and Yoshida 2010; Waidmann et al. 2014; Lang et al. 2015). DEK3 can bind histones and recruit topoisomerases. HMGB4 is part of a conserved family (within plants and animals) of chromatin‐associated proteins facilitating transcription (Bianchi and Agresti 2005). Although the exact role of HMGB4 remains unclear, this family is abundant and its members can bind Histone 1 linker sites. MBD10 is a methylated‐DNA reader and its family has been characterized to link methylated‐DNA and other histone PTMs that influence chromatin structure (Ravi et al. 2025). No previous studies have linked these components to the regulation of greening and the establishment of photosynthesis. Our work indicates that MBD10, DEK3 and HMGB4 act as putative negative regulators of *PhANGs*.

On the other hand, the *atpds5a*, *chr11*, and *chr17* lines showed reduced expression levels of *PORA* and *PORB* (Figure 5B), which correlated with reduced accumulation of photosynthetic pigments (Figure 5A). In addition, the c*hr11* and *chr17* lines showed reduced expression of *RBSC1B*, encoding the large subunit of Rubisco. Thus, the *atpds5a*, *chr11*, and *chr17* lines show consistently reduced accumulation of chlorophyll, impaired cotyledon opening and reduced *PhANG* expression during the greening process (Figures 4, 5, and S7). ATPDS5A is associated with cohesins which, in mammals, have been widely described as mediators of promoter‐enhancer loop formation (Kagey et al. 2010). In plants, cohesins have been linked to the creation of genomic fountains, which are related to high‐order genomic architecture (Wang et al. 2024). CHR11/17 mediate nucleosome sliding in the gene body and they function as subunits of the SWR1 complex to mediate H2A.Z deposition (Luo et al. 2020). Taken together, we identified ATPSD5A and CHR11/17 as H3K27ac interactors with an impact on *PhANG* expression and chlorophyll biosynthesis. Thus, our data suggest that these chromatin remodeling and enhancer‐promoter looping factors have a function as putative regulators of the establishment of photosynthesis.

## 4 Discussion

Our study establishes ChIP‐MS as a powerful new approach in plant chromatin biology, enabling the unbiased identification of proteins associated with specific histone modifications. By applying this method to H3K27ac, a mark central to gene activation, we uncovered a diverse set of candidates, including chromatin remodelers, histone variant‐associated factors, DNA‐binding proteins, and epigenetic readers. This reverse strategy—starting from a histone modification rather than a candidate factor—broadens the scope of discovery and highlights histone marks as organizational hubs that recruit multiple regulatory layers.

Several of the candidates identified here expand the current landscape of plant chromatin regulators. During the establishment of photosynthesis, chloroplast maturation is linked to histone acetylation and transcriptional activation of *PhANGs*. Besides general H3K27ac occupancy, we found that several of the H3K27ac ChIP‐MS interactors bind at *PhANG* loci (Figure S8). We also observed phenotypes related to the greening process for many of our candidates. For instance, the ISWI‐type remodelers CHR11 and CHR17 and the cohesin‐associated ATPDS5A emerged as positive regulators of chlorophyll biosynthesis and *PhANG* expression, suggesting that H3K27ac regions may require nucleosome positioning and long‐range chromatin interactions during chloroplast biogenesis. Conversely, MBD10, DEK3, and HMGB4 displayed phenotypes consistent with negative regulation of *PhANGs*, suggesting cross‐talk between acetylation, DNA methylation readers, and chromatin structural proteins. MBD10 has been suggested to be linked to heterochromatin (Preuss et al. 2008) and DEK3 to be acting both as an activator or repressor (Brestovitsky et al. 2019), suggesting that these factors may be playing a deactivation role for the H3K27ac regions. Together, these results illustrate the potential of this methodology to uncover new regulators associated with histone modifications, with broad implications for plant development and stress adaptation.

Despite these advances, the approach is not without caveats. First, the choice of crosslinkers (EGS plus formaldehyde) shapes the interactome recovered. While EGS effectively stabilizes more extended complexes, it may underrepresent direct or transient interactions, potentially biasing the protein list. In addition, the selection of antibodies is a critical determinant of success, as cross‐reactivity, epitope masking, or low affinity can limit the recovery of true interactors. Stringent proteomic thresholds reduce false positives but may have excluded low‐abundance regulators of functional significance, and mass spectrometry itself is biased toward abundant proteins, potentially overshadowing rare but biologically critical interactors. Diverse plant material could yield unique protein candidates specific to tissues or developmental stages. However, reducing the sample homogeneity by a mixed cell population can severely impact the experimental background. In our hands, increasing the amount of starting material above 50 g only resulted in more background (reflected in replicate 2, Figure 2A). Depending on the tissue, purity of the nuclei, and immunoprecipitation efficiency, further optimization could be achieved by using less starting material. Although the present protocol has been validated for Arabidopsis green tissue, specialized nuclei isolation methods for challenging tissues (e.g., those with high lignin or starch content) could be incorporated into the initial steps to obtain crosslinked chromatin. An exciting option would be to pair the enrichment of biotin‐tagged cell‐type‐specific nuclei (by INTACT, Benstein et al. 2023) with our ChIP‐MS protocol.

Crosslinking may also introduce artifacts by stabilizing indirect associations, and because ChIP‐MS provides only a static snapshot, it cannot capture the full dynamics of chromatin–protein interactions. Finally, functional validation based on mutant phenotypes demonstrates biological relevance but does not establish direct mechanistic roles in H3K27ac deposition or turnover. Nevertheless, our work demonstrates the feasibility of applying ChIP‐MS to plants and provides a first set of candidate regulators linked to H3K27ac deposition during the establishment of photosynthesis. The approach is readily extendable to other histone modifications (e.g., H3K4me3, H3K9me2) and histone variants (e.g., H2A.Z, H3.3), offering a systematic framework to dissect the protein environment of chromatin features in plants. By adapting ChIP‐MS protocols to the unique challenges of plant systems, we unlock the possibility of exploring plant chromatin with the same proteomic depth as animal systems, accelerating the discovery of epigenetic pathways that connect chromatin dynamics to development and environmental resilience.

## Author Contributions

M.Q., A.B., E.P.H., and Å.S. conceptualized the research, analyzed data, and wrote the paper. A.B., L.A.S., D.H.W.D., J.D., and M.Q. performed the experiments. All authors read and approved the final version of the manuscript.

## Funding

This work was supported by Stiftelsen för Strategisk Forskning (ARC19‐0051).

## Disclosure

AI‐tools were not used in any way in this manuscript.

## Supporting information


**Figure S1:** Polyacrylamide gels for Replicate 1 and Replicate 2. Polyacrylamide gels stained with Coomassie blue. The symbol * indicates the band specific to H3K27ac. Sliced gel pieces were processed for MS analysis.
**Figure S2:** Genotyping of T‐DNA insertion mutants of ChIP‐MS candidate proteins. Representative agarose gels from PCR‐based genotyping of the lines ordered from NASC and are SALK, Sail or GABI insertion lines. Table S2 for primer sequences. For each PCR primer combination, a Col0 genomic DNA template was used.
**Figure S3:** Description of fast screening method for chlorophyll accumulation. Detailed description of the procedure with representative pictures of the different steps.
**Figure S4:** ImageJ (.ijm) Macro Script for batch treatment of photos. This code has been created using the macro editor in ImageJ(Fiji) software. It allows the automatization of picture pre‐treatment before acquisition of grey values of the green channel. This entrusts that all pictures are prepared the same way while decreasing treatment time for users. The script sets brightness (min = 50 and max = 150), splits colour channels and closes blue and red channels to keep the green (grey values) channel of every picture. It finally closes original RGB pictures to avoid overwriting of original pictures during the measurements.
**Figure S5:** Representative pictures of cotyledons greening and opening in response to light. Representative images of 3‐day‐old seedlings grown in dark exposed to 6, 12 and 24 h constant white light. Scale bar 0.5 mm (Col0 24 h). Pictures have been acquired with an Epson Scanner 12000XL and analyzed with ImageJ (Fiji) for green pixel values.
**Figure S6:** Quantification of photosynthetic pigments. Independent additional experiments for pigment quantifications. Chlorophyll A, chlorophyll B, and total carotenoid concentrations were extracted with cold methanol and quantified by spectrophotometry from mutant candidates, and Col0 seedlings de‐etiolated during 12 h (*n* = 3 independent biological replicates). Significance was assessed using ordinary one‐way ANOVA comparisons followed by Dunnet's post hoc test for each mutant line against Col0 (*p*‐value thresholds: *p* < 0.05 (*), < 0.01 (**), < 0.001 (***)).
**Figure S7:** Analysis of cotyledon opening in response to light. 3‐day‐old seedlings grown in dark were exposed to 6, 12 and 24 h constant white light. Statistical analysis has been performed by a Two‐Way Anova test followed by a Dunnet's post hoc test (*p*‐value thresholds: *p* < 0.05 (*), < 0.01 (**), < 0.001 (***)) (n is comprised between min 44 and max 95 independent seedlings for each genotype). The background grey line is a help for readers (aligned to Col0 levels). **B**. The opening angle of the cotyledons has been measured with ImageJ using the Angle tool. A first line is traced along the hypocotyl, with the top of the angle orientated upward, a second line is traced to separate symmetrically both cotyledons. Only the seedlings presenting both cotyledons along the plane of the media have been selected to measure the angle.
**Figure S8:** ChIP‐MS candidates bind specific *PHANG* loci. **A**. Chromatin Immunoprecipitation (ChIP)‐sequencing(seq) visualization tracks of histone PTMs occupancy and H3K27ac ChIP‐MS interactors at 3 *PhANGs* evaluated during phenotyping in this study. A normalized ChIP‐seq signal is indicated on the y‐axis. A scale bar is indicated.

**Table S1:** Proteins identified from the ChIP‐MS analysis.

**Table S2:** Primers for genotyping T‐DNA insertion lines.

**Table S3:** Primers for qRT‐PCR.

## Data Availability

All raw data will be available upon request.
