# Supplementary material for: ChIP‐MS in Plant Systems: Mapping the H3K27ac Proteome During the Greening Process
Source: Physiol Plant. 2026 Feb 18;178(1):e70797. doi: 10.1111/ppl.70797 (PMC12915887; doi:10.1111/ppl.70797)
Supplement: Supplementary file 1 — Figure S1: Polyacrylamide gels for Replicate 1 and Replicate 2. Polyacrylamide gels stained with Coomassie blue. The symbol * indicates the band specific to H3K27ac. Sliced gel pieces were processed for MS analysis. Figure S2: Genotyping of T‐DNA insertion mutants of ChIP‐MS candidate proteins. Representative agarose gels from PCR‐based genotyping of the lines ordered from NASC and are SALK, Sail or GABI insertion lines. Table S2 for primer sequences. For each PCR primer combination, a Col0 genomic DNA template was used. Figure S3: Description of fast screening method for chlorophyll accumulation. Detailed description of the procedure with representative pictures of the different steps. Figure S4: ImageJ (.ijm) Macro Script for batch treatment of photos. This code has been created using the macro editor in ImageJ(Fiji) software. It allows the automatization of picture pre‐treatment before acquisition of grey values of the green channel. This entrusts that all pictures are prepared the same way while decreasing treatment time for users. The script sets brightness (min = 50 and max = 150), splits colour channels and closes blue and red channels to keep the green (grey values) channel of every picture. It finally closes original RGB pictures to avoid overwriting of original pictures during the measurements. Figure S5: Representative pictures of cotyledons greening and opening in response to light. Representative images of 3‐day‐old seedlings grown in dark exposed to 6, 12 and 24 h constant white light. Scale bar 0.5 mm (Col0 24 h). Pictures have been acquired with an Epson Scanner 12000XL and analyzed with ImageJ (Fiji) for green pixel values. Figure S6: Quantification of photosynthetic pigments. Independent additional experiments for pigment quantifications. Chlorophyll A, chlorophyll B, and total carotenoid concentrations were extracted with cold methanol and quantified by spectrophotometry from mutant candidates, and Col0 seedlings de‐etiolated during 12 h (n = 3 in [file PPL-178-e70797-s003.pdf]

SUPPLEMENTAL FIGURES & TABLES

Supplemental Figure 1. Polyacrylamide gels for Replicate 1 and Replicate 2.

Polyacrylamide gels stained with Coomassie blue. The symbol * indicates the band specific to H3K27ac. Sliced gel pieces were processed for MS analysis.

Supplemental Figure 2. Genotyping of T-DNA insertion mutants of ChIP-MS candidate proteins.

Representative agarose gels from PCR-based genotyping of the lines ordered from NASC and are SALK, Sail or GABI insertion lines. Supplemental Table 2 for primer sequences. For each PCR primer combination, a Col0 genomic DNA template was used.

Supplemental Figure 3. Description of fast screening method for chlorophyll accumulation.

Detailed description of the procedure with representative pictures of the different steps.

Supplemental Figure 4. ImageJ (.ijm) Macro Script for batch treatment of photos.

This code has been created using the macro editor in ImageJ(Fiji) software. It allows the automatization of picture pre-treatment before acquisition of grey values of the green channel. This entrusts that all pictures are prepared the same way while decreasing treatment time for users. The script sets brightness (min = 50 and max = 150), splits colour channels and closes blue and red channels to keep the green (grey values) channel of every picture. It finally closes original RGB pictures to avoid overwriting of original pictures during the measurements.

Supplemental Figure 5. Representative pictures of cotyledons greening and opening in response to light.

Representative images of 3-day-old seedlings grown in dark exposed to 6, 12 and 24h constant white light. Scale bar 0.5 mm (Col0 24h). Pictures have been acquired with an Epson Scanner 12000XL and analysed with ImageJ (Fiji) for green pixel values.

Supplemental Figure 6. Quantification of photosynthetic pigments.

Independent additional experiments for pigment quantifications. Chlorophyll A, chlorophyll B, and total carotenoid concentrations were extracted with cold methanol and quantified by spectrophotometry from mutant candidates, and Col0 seedlings de-etiolated during 12 h (n=3 independent biological replicates). Significance was assessed using ordinary one-way ANOVA

comparisons followed by Dunnet's post hoc test for each mutant line against Col0 (p-value thresholds: p <0.05 (*), <0.01(**), <0.001(***).

Supplemental Figure 7. Analysis of cotyledon opening in response to light.
3-day-old seedlings grown in dark were exposed to 6, 12 and 24h constant white light. Statistical analysis has been performed by a Two-Way Anova test followed by a Dunnet's posthoc test (p-value thresholds: p <0.05 (*), <0.01(**), <0.001(***)) (n is comprised between min 44 and max 95 independent seedlings for each genotype). The background grey line is a help for readers (aligned to Col0 levels). **B.** The opening angle of the cotyledons has been measured with ImageJ using the Angle tool. A first line is traced along the hypocotyl, with the top of the angle orientated upward, a second line is traced to separate symmetrically both cotyledons. Only the seedlings presenting both cotyledons along the plane of the media have been selected to measure the angle.

Supplemental Figure 8. ChIP-MS candidates bind specific PHANG loci.
A. Chromatin Immunoprecipitation (ChIP)-sequencing(seq) visualization tracks of histone PTMs occupancy and H3K27ac ChIP-MS interactors at 3 PhANGs evaluated during phenotyping in thisstudy. A normalized ChIP-seq signal is indicated on the y-axis. A scale bar is indicated.

Supplemental Table 1. Proteins identified from the ChIP-MS analysis
Supplemental Table 2. Primers for genotyping T-DNA insertion lines.
Supplemental Table 3. Primers for qRT-PCR.

Figure S1

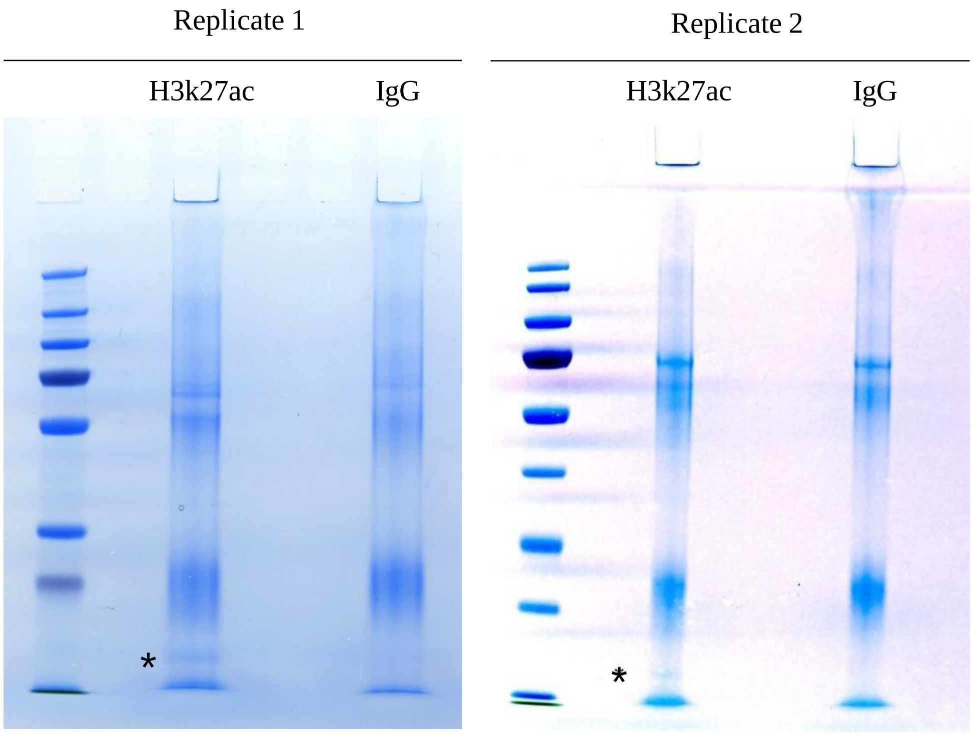

Figure S2

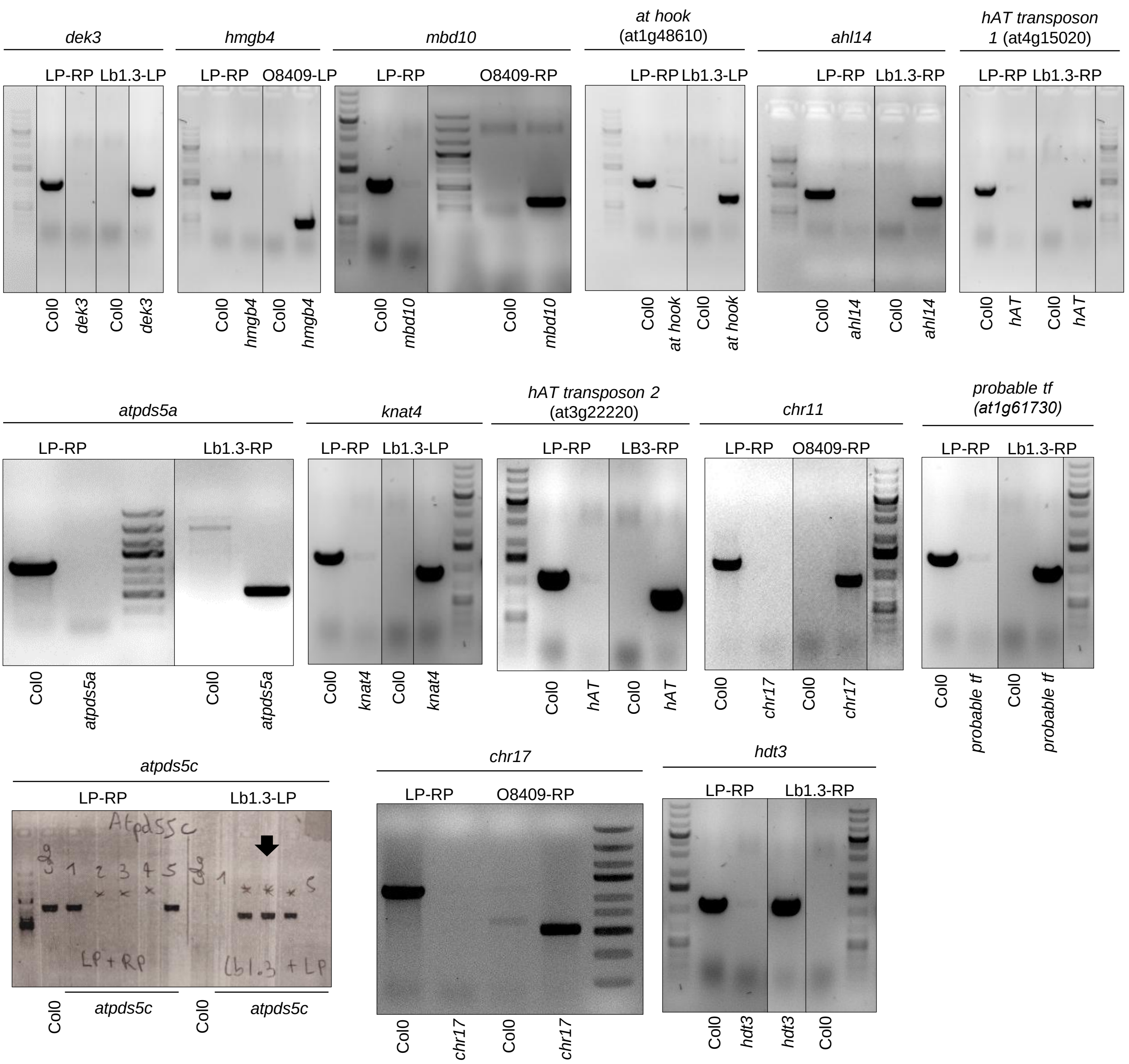

dek3
LP-RP Lb1.3-LP
Col0 dek3 Col0 dek3
hmgb4
LP-RP O8409-LP
Col0 hmgb4 Col0 hmgb4
mbd10
LP-RP O8409-RP
Col0 mbd10 Col0 mbd10
at hook (at1g48610)
LP-RP Lb1.3-LP
Col0 at hook Col0 at hook
ahl14
LP-RP Lb1.3-RP
Col0 ahl14 Col0 ahl14
hAT transposon 1 (at4g15020)
LP-RP Lb1.3-RP
Col0 hAT Col0 hAT
atpds5a
LP-RP Lb1.3-RP
Col0 atpds5a Col0 atpds5a
knat4
LP-RP Lb1.3-LP
Col0 knat4 Col0 knat4
hAT transposon 2 (at3g22220)
LP-RP LB3-RP
Col0 hAT Col0 hAT
chr11
LP-RP O8409-RP
Col0 chr17 Col0 chr17
probable tf (at1g61730)
LP-RP Lb1.3-RP
Col0 probable tf Col0 probable tf
atpds5c
LP-RP Lb1.3-LP
Col0 atpds5c Col0 atpds5c
chr17
LP-RP O8409-RP
Col0 chr17 Col0 chr17
hdt3
LP-RP Lb1.3-RP
Col0 hdt3 hdt3 Col0

Figure S3

a. Acquiring pictures

Plates are scanned using a scanner with manual settings and manual focus available. The advantage of a scanner, instead of a camera is the total control and reproducibility of the light parameters and white balance. Those are essential for repeatability of experiments and their comparison over time; knowing how to control light sources and white balance are complex skills to learn when using a camera : anyone can use scanner settings. Plates are scanned lid open and through the media. The focus is set manually to optimize the scan on the seedlings. Pictures are saved as TIFF format and treated with ImageJ (FIJI) software.

e.g. Epson Scanner 12000XL

High resolution 24bit RGB .TIFF

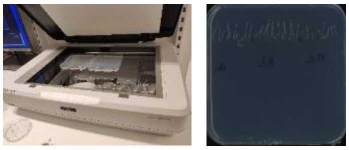

b. Adjust Brightness

After acquisition, all pictures have their Brightness adjusted at the same values. The histogram is set at min = 50 and max = 150.

Adjusted pictures

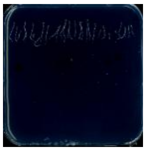

c. Split RGB channels

To track only the green value of seedlings, we split color channels of pictures to obtain three sub pictures each a 8-bit grey value picture showing its respective color hue. Here only the green channel interests us, the red and blue channels are discarded

Color channels

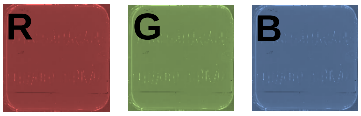

d. Draw ROI and measure grey pixel value

On each seedling « aligned » with the plan of the media, a ROI is drawn on one cotyledon. After all the correct seedlings of the same genotype have a ROI, means of grey pixel value of each ROI are measured. On a 8-bit format picture all grey pixel values are distributed from 0 to 255 with the minimum 0 being total black and the maximum 255 being a total white. If the grey value diminishes, the color intensity increases. So to compare linearilythe color variation we invert all measurement by substrating the grey value to the maximum 255. The same pictures are used for cotyledon opening angle.

Green channel (grey values)

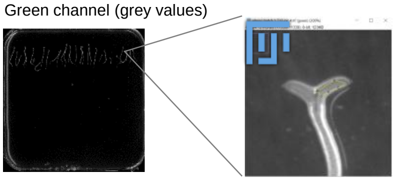

Figure S4

```
// Get a stable list of the titles of all currently open images
titles = getList("image.titles");
n = titles.length;

// Process each original image
for (i = 0; i < n; i++) {
    // Work on the i-th originally open image
    if (!isOpen(titles[i])) continue; // in case it was closed earlier
    selectWindow(titles[i]);

    imageName = getTitle();

    // Set brightness/contrast
    setMinAndMax(50, 150);

    // Save a copy to Desktop
    desktopPath = getDirectory("home") + "Desktop/";
    saveAs("Tiff", desktopPath + imageName);

    wait(2500); // time break adjust or remove if not needed depending on your processor

    // Check whether the image is multichannel
    getDimensions(w, h, channels, slices, frames);
    isRGB = bitDepth == 24;
    isMulti = (channels > 1) || isRGB;

    if (isMulti) {
        // Duplicate so we don't break our loop
        run("Duplicate...", "title=__WORK__");
        wait(200);

        selectWindow("__WORK__");
        run("Split Channels");
        wait(500);

        // Close the duplicate source
        if (isOpen("__WORK__")) { selectWindow("__WORK__"); close(); }

        // Close red & blue channels
        if (isOpen("__WORK__ (red)"))  { selectWindow("__WORK__ (red)");  close(); }
        if (isOpen("__WORK__ (blue)")) { selectWindow("__WORK__ (blue)"); close(); }

        // Rename green channel to match original
        if (isOpen("__WORK__ (green)")) {
            selectWindow("__WORK__ (green)");
            rename(imageName + " (green)");
        }
    } else {
        print("Skipping split for \"" + imageName + "\" (not multichannel).");
    }
}

// After processing all, close the original RGB images
for (i = 0; i < n; i++) {
    if (isOpen(titles[i])) {
        selectWindow(titles[i]);
        close();
    }
}
```

Figure S5

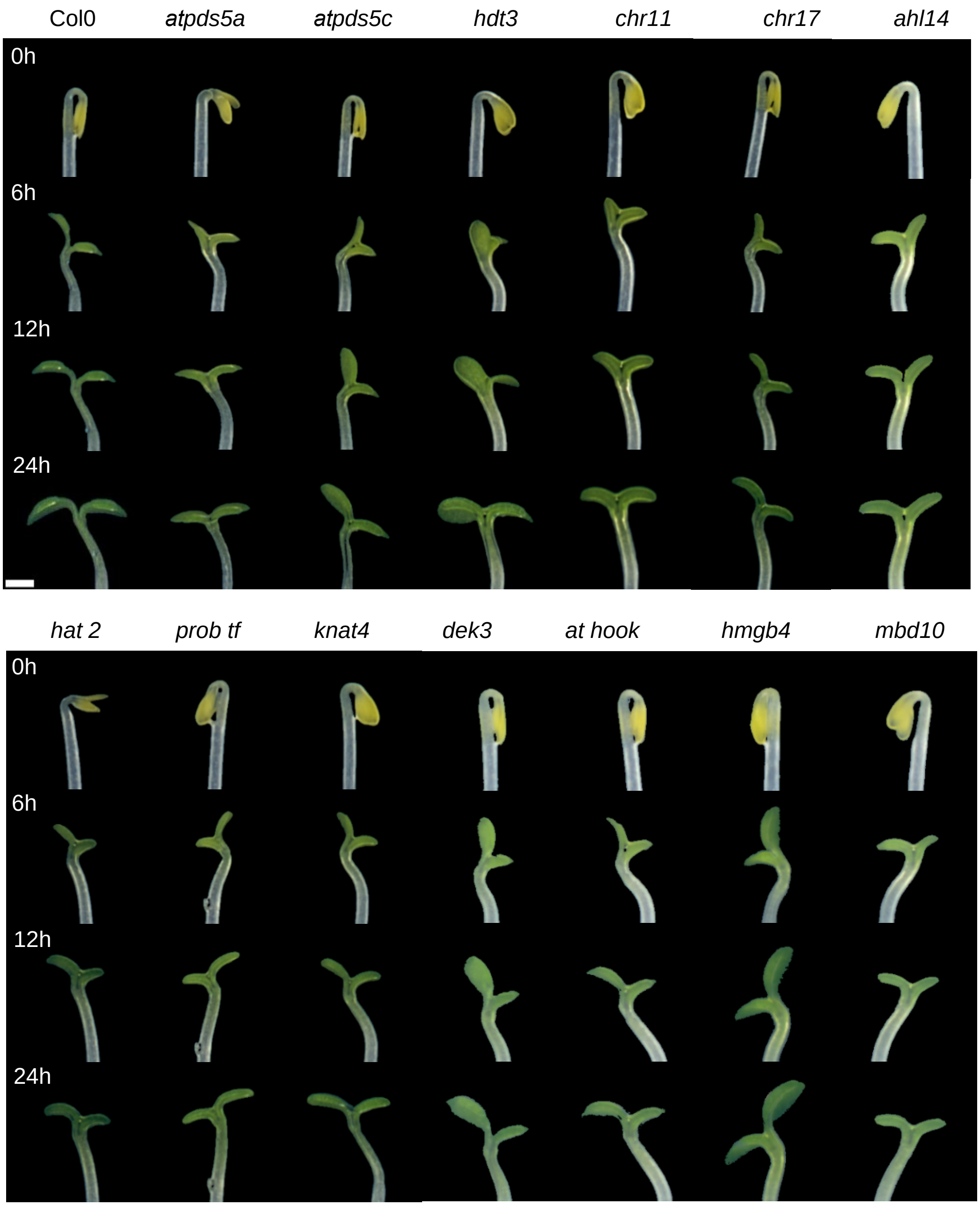
Col0
atpds5a
atpds5c
hdt3
chr11
chr17
ahl14
0h
6h
12h
24h
hat 2
prob tf
knat4
dek3
at hook
hmgb4
mbd10
0h
6h
12h
24h

Figure S6

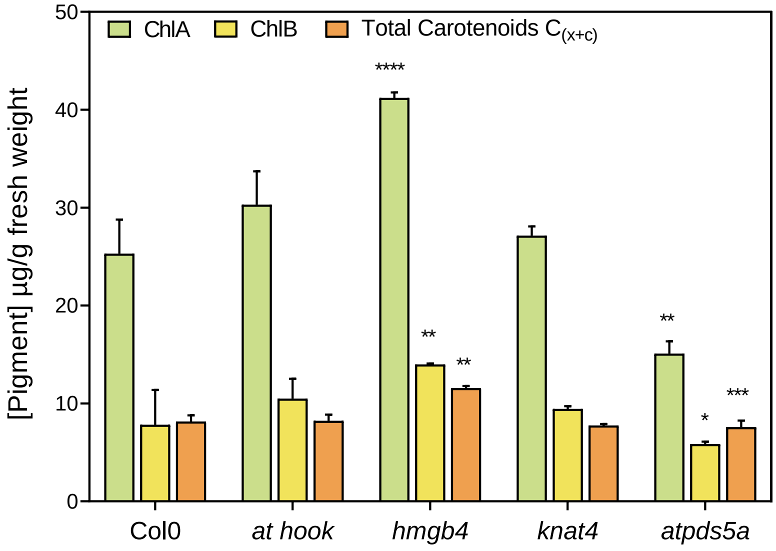

ChlA
ChlB
Total Carotenoids C(x+c)
[Pigment] µg/g fresh weight
50
40
30
20
10
0
****
**
**
**
*
***
Col0
at hook
hmgb4
knat4
atpds5a

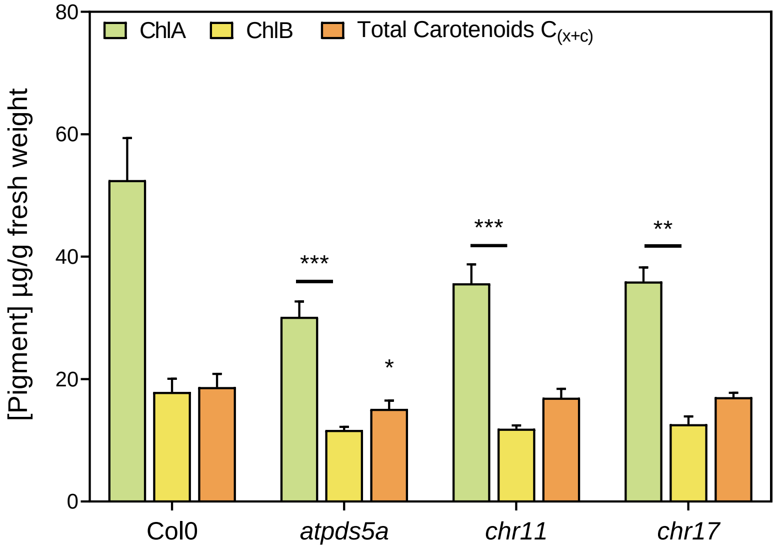

ChlA
ChlB
Total Carotenoids C(x+c)
[Pigment] µg/g fresh weight
80
60
40
20
0
***
*
***
**
Col0
atpds5a
chr11
chr17

Figure S7

A.

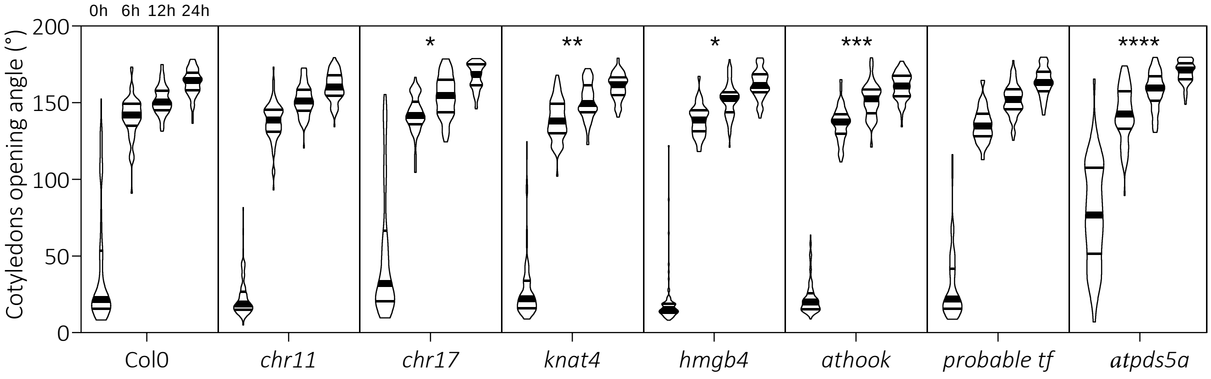

0h 6h 12h 24h
Cotyledons opening angle (°)
200
150
100
50
0
*
**
*
***
****
Col0
chr11
chr17
knat4
hmgb4
athook
probable tf
atpds5a

B.

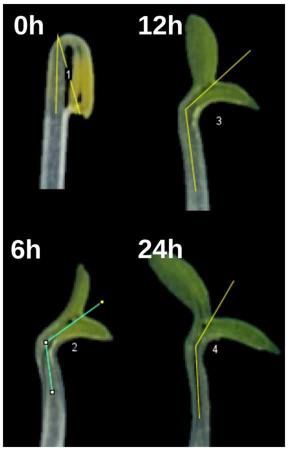

0h
12h
6h
24h
1
2
3
4

Figure S8

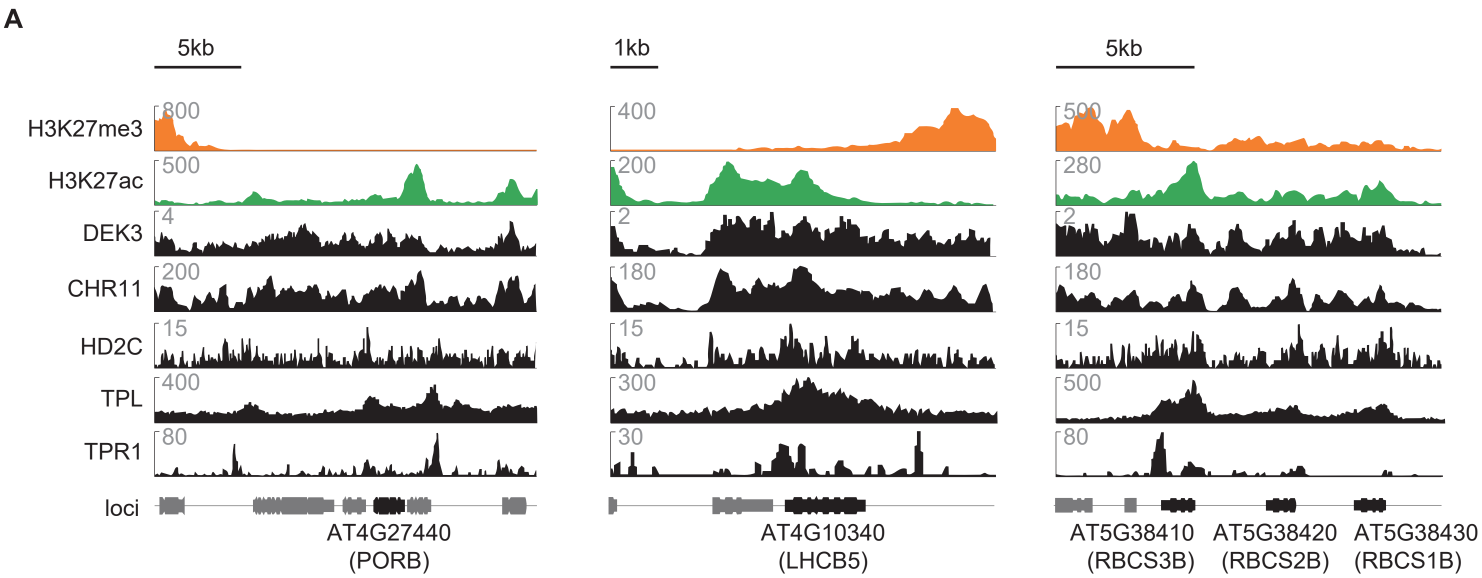
A
5kb
1kb
5kb
H3K27me3
H3K27ac
DEK3
CHR11
HD2C
TPL
TPR1
loci
800
500
4
200
15
400
80
400
200
2
180
15
300
30
500
280
2
180
15
500
80
AT4G27440
(PORB)
AT4G10340
(LHCB5)
AT5G38410
(RBCS3B)
AT5G38420
(RBCS2B)
AT5G38430
(RBCS1B)
